# Pediatric low-grade epilepsy-associated tumors (LEATS): neuroimaging review and genetics update

**DOI:** 10.1007/s00247-026-06519-z

**Published:** 2026-01-27

**Authors:** Lindsey Pelissier, Asha Sarma, Joanne Rispoli, Stephen Little, Sumit Pruthi, Alexandra Foust

**Affiliations:** 1 Monroe Carell Jr. Children’s Hospital at Vanderbilt, 2200 Children’s Way, STE 1421, Nashville, TN 37232-9700 USA; 2 Boston Children’s Hospital, Boston, MA USA; 3 Children’s Healthcare of Atlanta, Atlanta, GA USA

**Keywords:** Brain neoplasms, Central nervous system, Child, Epilepsy, Mutation

## Abstract

Low-grade epilepsy-associated tumors (LEATs) are a distinct group of tumors commonly encountered in pediatric drug-resistant epilepsy that necessitate surgical intervention. Like tumors elsewhere in the central nervous system, molecular characterization is becoming an increasingly important consideration in pediatric neuro-oncology prognostication and management for LEATs. Thus, familiarity with relevant tumor mutations and radiogenomic features of LEATs is important for radiologists caring for affected patients. This article will review the genetic alterations and imaging characteristics of LEATs, formatted according to the three categories defined by the World Health Organization (WHO): glioneuronal and neuronal tumors (ganglioglioma, dysembryoplastic neuroepithelial tumor, papillary glioneuronal tumor, multinodular and vacuolating neuronal tumor); circumscribed astrocytic gliomas (pilocytic astrocytoma, pleomorphic xanthoastrocytoma); and pediatric-type diffuse low-grade gliomas (diffuse astrocytoma *MYB or MYBL1*-altered, angiocentric glioma, diffuse low-grade glioma MAPK pathway-altered, polymorphous low-grade neuroepithelial tumor of the young).

**Graphical abstract:**

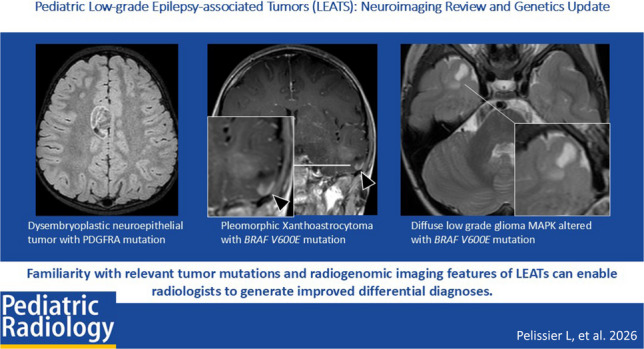

## Introduction

The term LEAT (low-grade (or long-term per some sources) epilepsy-associated tumors), first introduced by Luyken et al. in 2003, has been used to describe a distinct group of tumors commonly presenting with early-onset (childhood), drug-resistant epilepsy [1]. Although somewhat histologically diverse, most LEATs are WHO grade 1 tumors (low-grade gliomas can also be WHO grade 2) and are generally considered neuroepithelial tumors, composed of mixed glial and neuronal cell components. These tumors typically present in childhood (average age of 13–14 years old) and have a predilection for the temporal lobes [2, 3]. Surgical resection can lead to seizure reduction or complete seizure freedom in >80% of patients. Importantly, a longer duration of epilepsy related to LEATs has been associated with unfavorable post-surgical seizure outcomes and lower full-scale intelligence quotient; thus, early identification is important [4, 5].

As emphasized in the 2021 World Health Organization’s central nervous system (CNS) tumor classification update, molecular characterization is becoming an increasingly important component of pediatric neuro-oncology, particularly given the continued development of molecularly targeted treatment options. Thus, familiarity with relevant tumor mutations and radiogenomic features can enable radiologists to generate more accurate differential diagnoses. Unlike in many adult gliomas, *IDH1* mutation, 1p/19q co-deletion, *TERT* promoter mutations, and *MGMT DNA* methylation are not commonly implicated in LEATs [2]. Instead, commonly encountered genetic alterations in LEATs include *BRAF V600E* (predominated by ganglioglioma-like alterations), *FGFR1/FGFR2* (predominated by oligodendrocyte-like cells), and *MYB* (astrocytic and angiocentric patterns) mutations [6]. LEAT entities fall into three distinct categories within the “Gliomas, glioneuronal tumors, and neuronal tumors” family under the WHO 5th edition 2021 CNS tumor classification system: glioneuronal and neuronal tumors, circumscribed astrocytic gliomas, and pediatric-type diffuse low-grade gliomas. Table 1 demonstrates tumor entities within each of these groups, their relevant genetic alterations, and key imaging features. While gross total resection remains the mainstay of treatment for most LEATs, targeted chemotherapy agents such as *BRAF* or *MEK* inhibitors may have an increasing role in the future for patients who are poor surgical candidates, have incomplete resections, or have higher risk for recurrence [7, 8]. Post-operative monotherapy with BRAF inhibitors can be effective in pediatric pilocytic astrocytoma, ganglioglioma, pleomorphic xanthoastrocytoma, and pediatric low-grade glioma tumors harboring the *BRAF*^*V600E*^ mutation [9].

**Table 1:** Common genetic variants and key neuroimaging features of LEATs

Category	Type	Common genetic alterations	Key neuroimaging features	Differential diagnosis
Glioneuronal and neuronal tumors	Ganglioglioma	BRAF	- Ca++ common - May exhibit low diffusivity - Solid or solid and cystic, often with nodular or rim enhancement	DNT, focal cortical dysplasia (FCD), PGNT, Pilocytic or DLGG-MAP pathway-altered, PLNTY, PXA
	Dysembryoplastic neuroepithelial tumor (DNT)	FGFR1, PDGFRA, BRAF	- Multi-cystic, “bubbly” appearing cortically based tumor - Margins may be indistinct when assoc. w/cortical dysplasia - May have T2-FLAIR mismatch sign	Ganglioglioma, FCD, MVNT, angiocentric glioma
	Papillary glioneuronal tumor (PGNT)	SLC44A1-PRKCA	- T2-heterogeneously hyperintense - Strong, heterogeneous enhancement	Ganglioglioma, pilocytic astrocytoma, ependymoma
	Multinodular and vacuolating neuronal tumor (MVNT)	MAPK pathway	- Cluster of variably sized, discrete T2-/FLAIR-hyperintense nodules - Located at the gray-white junction, depth of sulcus	DNT, FCD, prominent/atypical perivascular spaces
Circumscribed astrocytic gliomas	Pilocytic astrocytoma	KIAA 1549-BRAF fusion, BRAF, NF-1	- Enhancing mural nodule with cyst - Minimal surrounding edema	Ganglioglioma, ependymoma, PXA
	Pleomorphic xanthoastrocytoma (PXA)	BRAF-CDKN2A/B	- Enhancing nodule abutting pia often with adjacent meningeal enhancement	Ganglioglioma, DNT, pilocytic astrocytoma
Pediatric-type diffuse low-grade gliomas	Diffuse astrocytoma	MYB and MYBL1	- T2/FLAIR-heterogeneously hyperintense - May appear infiltrative - Non-enhancing	DLGG MAPK pathway-altered, DNT, angiocentric glioma, astrocytoma-IDH mutant
	Angiocentric glioma	MYB-QKI fusion	- May have rim-like T1 hyperintensity - T2/FLAIR hyperintense with blurred gray-white interface - May have “stalk-like” extension toward lateral ventricle	Diffuse astrocytoma, *MYB* or *MYBL1*-altered, FCD, DNT, ganglioglioma, PLNTY, astrocytoma-IDH mutant
	Diffuse low-grade glioma (DLGG), MAPK pathway-altered	FGFR1 BRAF TKD	- Infiltrating T1 mixed, T2/FLAIR hyperintense - Variable enhancement	Pilocytic astrocytoma, ganglioglioma, DNT, diffuse astrocytoma *MYB* or *MYBL1*-altered, astrocytoma-IDK mutant, PLNTY
	Polymorphous low-grade neuroepithelial tumor of the young (PLNTY)	BRAF and FGFR family (FGFR2 fusion, FGFR3)	- Well-circumscribed, cortically-based - Dense or bulky Ca++ - Non-enhancing	Oligodendroglioma, DNT, ganglioglioma, FCD, astrocytoma-IDK mutant, MVNT, calcifying pseudoneoplasm of the neuroaxis (CAPNON)

## Imaging technique

According to the International League Against Epilepsy, MRI of the brain per the Harmonized Neuroimaging of Epilepsy Structural Sequence (HARNESS) protocol should be performed soon after a pediatric patient presents with a first time seizure [10]. Notably, the HARNESS protocol specifically calls for high-resolution 3D T1-weighted, 3D FLAIR, and high in-plane resolution 2D coronal T2-weighted images. Three-tesla MRI is preferred; however, the protocol can also be performed on 1.5-T MRI systems depending on availability. The imaging protocol utilized at our institution is shown in Table 2. In cases of suspected neoplasm, post-contrast volumetric T1-weighted images are also acquired.

**Table 2:** Epilepsy imaging protocol

3 T	1.5 T
3D T1-weighted TFE (0.9 mm)	3D T1-weighted TFE (0.9 mm)
AX T2-weighted TSE (2.5 mm, gap 0)	AX T2-weighted TSE (2.5 mm, gap 0)
COR STIR TSE (2.5 mm, gap 0) (whole brain angled to temporal lobe)	COR STIR TSE (2.5 mm, gap 0) (whole brain angled to temporal lobe)
AX SWI (3 mm)	AX T2 FFE
AX DTI (4 mm)	AX DTI (4 mm)
3D T2 FLAIR (1 mm)	3D T2 FLAIR (1 mm)
3D ASL	Post-contrast 3D TFE T1 (0.9 mm)
Post-contrast 3D TFE T1 (0.9 mm)	Post-contrast 3D TSE T1 (0.9 mm)
Post-contrast 3D TSE T1 (0.9 mm)	

Alternative volumetric 3D T1-weighted sequences (e.g., MPRAGE, FFE, FGRE, fast SPGR) appropriate depending on vender. Axial DWI/ADC may be used in place of DTI. Post-contrast T1 FSE may alternatively be used

*TFE* turbo field echo, *TSE* turbo spin echo, *STIR* short tau inversion recovery, *SWI* susceptibility-weighted imaging, *DTI* diffusion tensor imaging, *FLAIR* fluid attenuation inversion recovery, *ASL* arterial spin labeling, *FFE* fast field echo

## General considerations in the neuroimaging of LEATs

While the spectrum of tumors under the LEAT umbrella is broad, it is important to note that gangliogliomas and dysembryoplastic neuroepithelial tumors (DNTs) account for more than three-quarters of cases [3, 11]. Therefore, gangliogliomas and DNTs are usually important differential diagnostic considerations when a LEAT is suspected on imaging. LEATs can be difficult to differentiate from focal cortical dysplasia (FCD) and can even co-occur with FCD type IIIb. The incidence of co-occurrence varies by tumor type and is most common with gangliogliomas and dysembryoplastic glioneuronal tumors (DNT) [12]. Lesions that have an expansile appearance, internal enhancement, or show interval growth should raise concern for some component of underlying neoplasia. In addition, low-grade gliomas in pediatric patients are more likely to be “dual pathology” lesions, or tumors that co-occur with secondary potentially epileptogenic findings such as mesial temporal sclerosis, gliosis, or vascular malformations (i.e., cavernous malformation), in addition to FCD IIIb. Therefore, it is important to pay close attention to the amygdala, hippocampal formations, and surrounding cortex and subcortical white matter when evaluating a patient with a suspected LEAT lesion. Surgical resection is often curative, with up to 75.9% demonstrating freedom from disabling seizures at 5 years post-surgery [11].

## Glioneuronal and neuronal tumors

### Ganglioglioma

Gangliogliomas represent the most common LEAT tumor, accounting for up to 37–44% of lesions in two large surgical cohorts [3, 11]. They have a propensity to arise in the temporal lobe, with up to 45% of pediatric cases presenting with a temporal lobe lesion, and can co-exist with focal cortical dysplasia [13, 14]. Gangliogliomas have a slight male predominance and have a peak age of presentation between 10–30 years [13, 15]. *BRAF* mutations are the most commonly encountered genetic alteration, with *BRAF V600E* mutations accounting for 40–50% of cases and *KIAA1549-BRAF* fusion representing another 10–15% of cases [16]. Surgical resection yields an excellent prognosis.

**Fig. 1:**
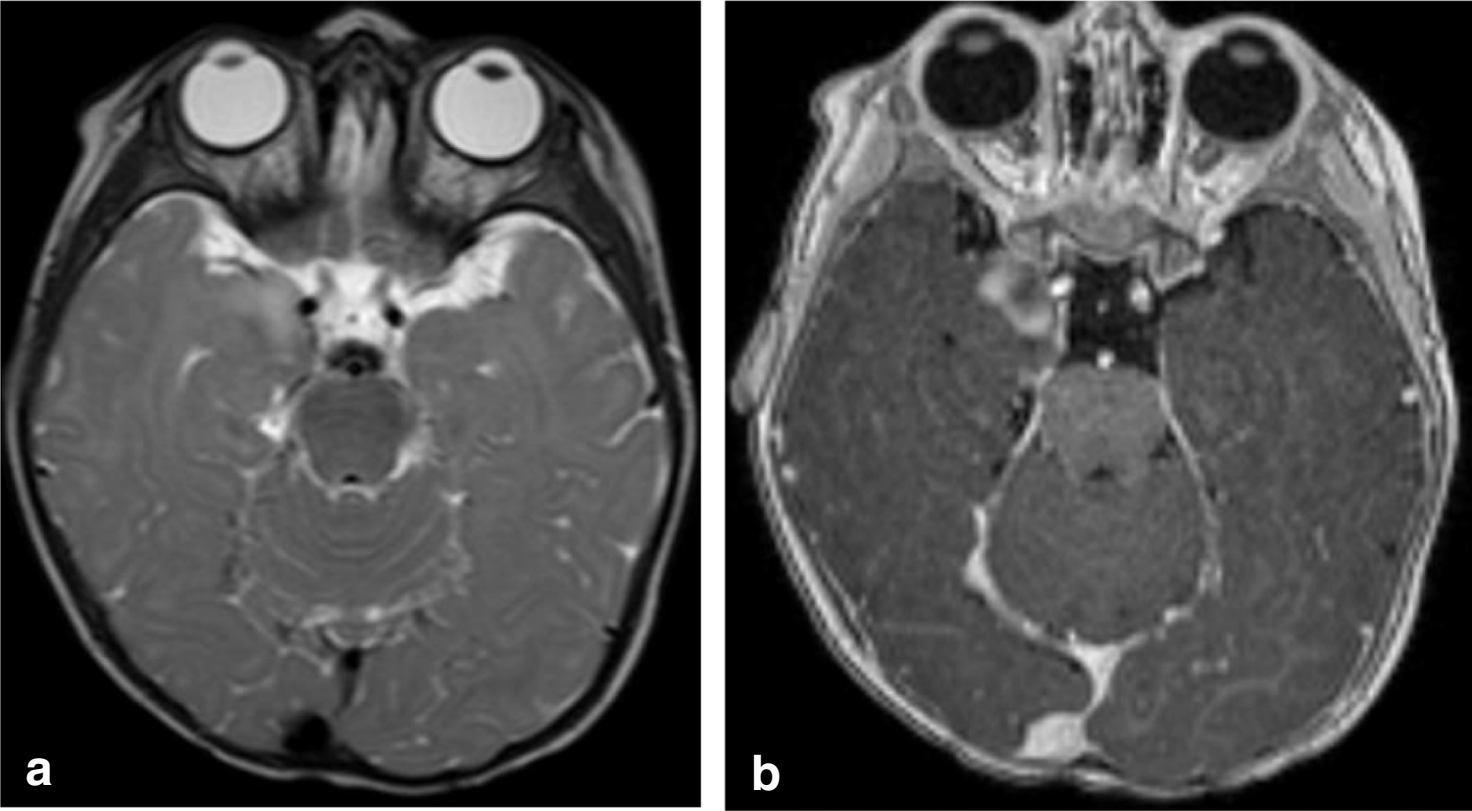
Ganglioglioma with *BRAF V600E* mutation in a 7-month-old male presenting with seizures. Axial T2-weighted (**a**) and T1-weighted contrast-enhanced (**b**) MR images show an ill-defined, mildly expansile, cortically-based right temporal lobe mass with nodular enhancement

In practice, gangliogliomas can demonstrate a wide range of imaging appearances; however, they generally fall into two dominant patterns: a circumscribed cyst with enhancing mural nodule or a solid tumor with variable enhancement that often expands the involved gyri (Fig. 1). Solid tumor components appear hypo- to isoattenuating on CT and demonstrate T1 hypo- to isointense signal and T2-hyperintense signal relative to cortex on MR imaging [17]. Interestingly, gangliogliomas tend to be larger and more cystic in appearance in younger patients and tumors with cystic components have been shown to have a higher likelihood of harboring a *BRAF V600E* mutation [18]. Calcifications may be seen in up to 35% of cases [19]. Surrounding vasogenic edema is often absent and intralesional hemorrhage is uncommon [20]. Of note, gangliogliomas can exhibit low diffusivity which can mimic higher grade lesions, with *BRAF V600E* mutant gangliogliomas showing lower ADC values than *BRAF V600E*-wild type tumors [19]. Ill-defined enhancing margins and more infiltrative appearance have also been more frequently reported in *BRAF V600E* mutant gangliogliomas than *BRAF V600E*-wild type tumors (Fig. 1) [21].

### Dysembryoplastic neuroepithelial tumor (DNT)

DNTs are the second most common LEAT type identified in epileptogenic tumor specimens, accounting for approximately 17–25% [2, 3]. Affected patients typically present in the second decade (peak age between 10–14 years) and there is a slight male predominance [22]. Similar to gangliogliomas, DNTs are most commonly encountered in the temporal lobes and can co-exist with focal cortical dysplasia [8, 14, 22]. *FGFR1* mutation occurs in up to 90%, while alterations in *BRAF V600E* are reported in 20–51% of cases [6, 22, 23]. Mutations in *PDGFRA* have also been reported and are particularly common in DNTs occurring along the septum pellucidum [24]. Malignant transformation is rare.

**Fig. 2:**
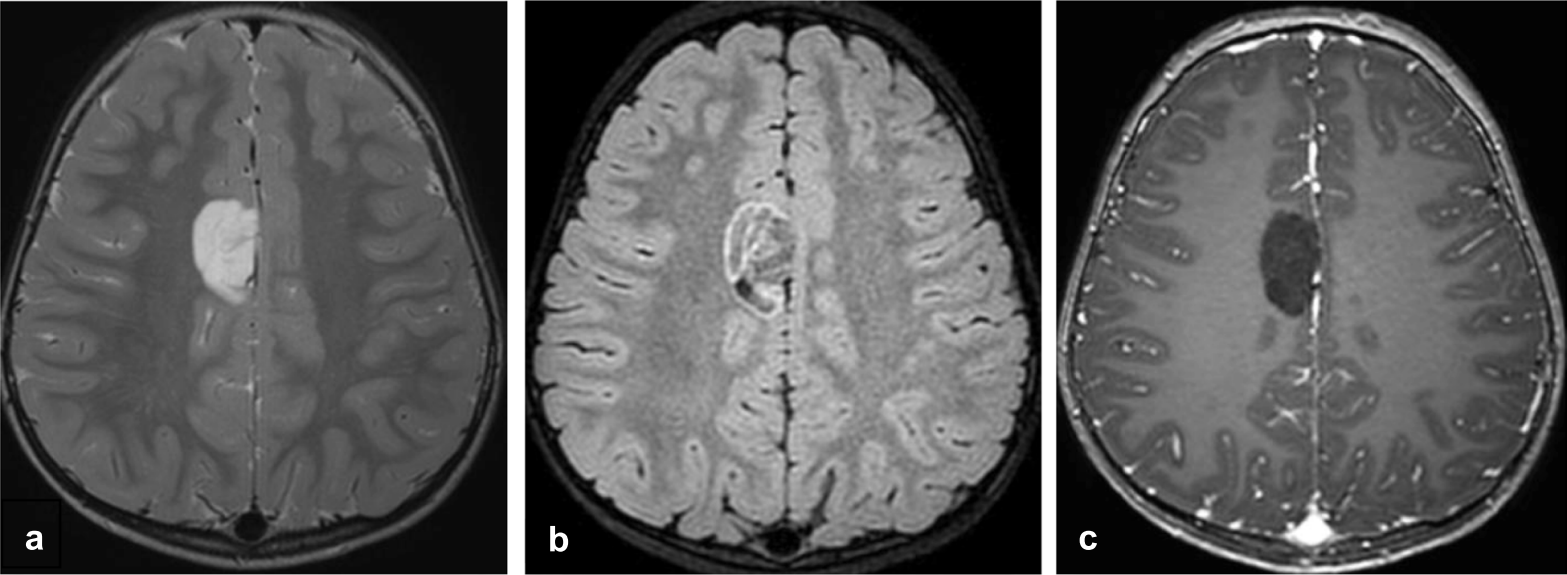
DNT with *PDGFRA* mutation in a 3-year-old female presenting with a seizure. Axial T2-weighted (**a**), T2 FLAIR (**b**), and T1-weighted contrast-enhanced (**c**) MR images demonstrate a “bubbly,” cortically-based, non-enhancing right paramedian frontal lobe mass with “T2-FLAIR mismatch” sign

Imaging typically reveals a wedge-shaped, well-circumscribed, “bubbly” multi-cystic, hypoattenuating and T2-hyperintense, cortically-based gyriform lesion with minimal surrounding edema [15, 25–27]. Nodular, less distinct lesions with blurring of the gray-white junction are a less common presentation [28]. On FLAIR imaging, DNTs generally suppress centrally with a FLAIR-hyperintense rim (“T2-FLAIR mismatch” sign) (Fig. 2), which can help distinguish DNTs from other low-grade gliomas, oligodendrogliomas, gangliogliomas, and multinodular and vacuolating neuronal tumors (MVNTs) [29–31]. That being said, in the adult population, the T2-FLAIR mismatch sign is nearly pathognomonic of *IDH*-mutant, *1p/19q* non-codeleted astrocytoma, which is distinguished by avid enhancement and a different genetic profile. DNTs usually show no enhancement on post-contrast imaging, although punctate or nodular enhancement can occur in approximately 20–30% of cases [22]. Intralesional calcifications may be observed in approximately one-third of cases [32]. DNTs lack diffusion restriction and often show hyperintense signal on ADC maps [29].

### Papillary glioneuronal tumor (PGNT)

PGNT is a rare WHO grade I tumor, formerly considered a subtype of ganglioglioma before being recognized as a distinct entity in 2007, with fewer than 150 cases reported in the literature [33]. The age at diagnosis is variable; however, the majority of cases present during the second and third decades and there is a 1.42:1 male to female ratio [33]*.* PGNTs occur most frequently in the frontal lobes, followed by the temporal lobes, often in a periventricular location [33, 34]. *SLC44A1-PRKCA* fusion is the most common genetic alteration [35]*.*

**Fig. 3:**
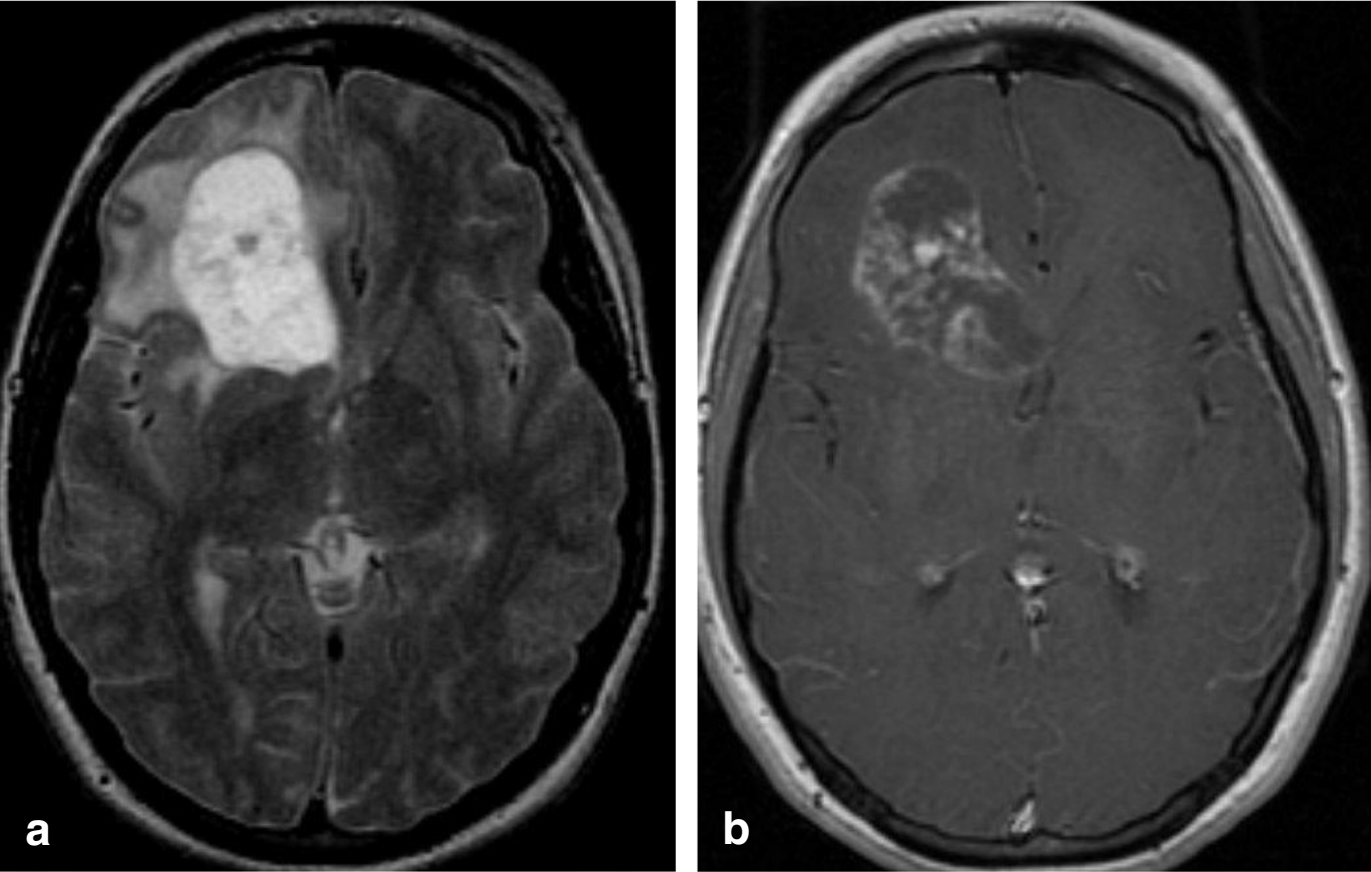
Papillary glioneuronal tumor (PGNT) in a 17-year-old female who presented with a seizure. Axial T2-weighted (**a**) and T1-weighted contrast-enhanced (**b**) MR images demonstrate a heterogeneous, predominantly T2-hyperintense right frontal lobe mass with nodular and stippled internal enhancement. Note the moderate degree of surrounding vasogenic edema

The imaging appearance of PGNTs is variable, ranging from a circumscribed cyst with an avidly enhancing mural nodule to a completely solid mass [36]. A heterogeneously enhancing solid and cystic lesion is most common (Fig. 3) [33]. The absence of diffusion restriction and low rCBV may help to differentiate these lesions from higher grade lesions [36]. Perilesional edema may be observed in approximately 25–46% of cases [33, 34].

### Multinodular and vacuolating neuronal tumor (MVNT)

MVNT was recognized as a distinct tumor type in the 2016 WHO classification for tumors of the CNS [37]. Unlike most LEAT lesions, MVNTs are more commonly encountered in adult patients, although the ages of reported presentation range from 6 years to 77 years [38]. They occur most commonly in the juxtacortical cerebral hemispheres, although can rarely occur in the cerebellum [39]. There is some debate in the literature about whether MVNT represents a malformation or a neoplasm; however, the adult presentation and molecularly confirmed alterations in the Ras-Raf-MAP kinase signaling pathway are supportive of neoplasia [40]. Furthermore, the most recent 2021 WHO classification also considers MVNT as a grade 1 tumor. Diagnosis is generally presumptive based on imaging alone in most cases of MVNT as most cases are followed with imaging rather than resected.

**Fig. 4:**
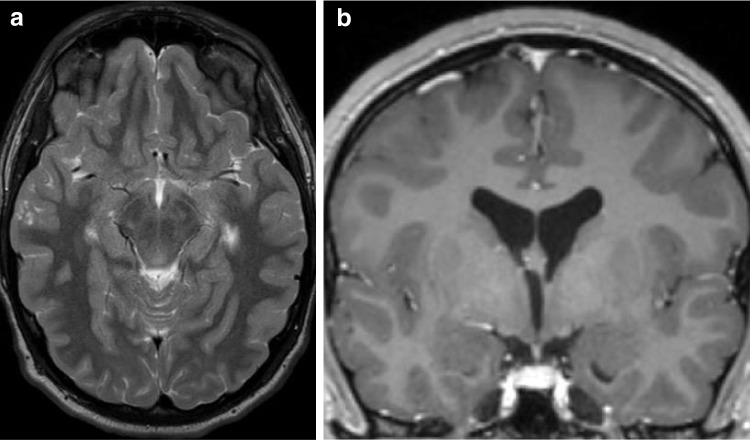
Multinodular and vacuolating neuronal tumor (MVNT) in a 15-year-old male who presented with a seizure. Axial T2-weighted (**a**) and coronal T1-weighted contrast-enhanced (**b**) MR images reveal tiny, clustered, ovoid, T2-hyperintense, non-enhancing foci located along the deep aspect of the cortical ribbon of the right temporal lobe

The characteristic imaging appearance of MVNT is a cluster of tiny (1–5 mm), ovoid, circumscribed T2-hyperintense lesions centered along the deep surface of the cortical ribbon and adjacent superficial white matter (Fig. 4) [41]. The degree of T2-hyperintensity tends to be less than the ventricles [41]. On FLAIR images, MVNTs demonstrate persistent hyperintense signal, which can help differentiate them from DNTs or prominent perivascular spaces [30, 41]. They are not associated with lesional contrast enhancement [37, 38, 41]. Diffusion-weighted images may show mild hyperintensity without corresponding ADC hypointensity [30, 38].

## Circumscribed astrocytic gliomas

### Pilocytic astrocytoma

Pilocytic astrocytoma is the most common primary pediatric brain tumor, accounting for 15.6% of all brain tumors, though the majority arise in the posterior fossa or involve the optic pathway [42]. Cerebral lesions are less common, though they are more likely to present with medically refractory seizures, particularly if located in the temporal lobes. Commonly observed genetic alterations include the KIAA 1549-BRAF fusion and NF1 germline mutations [43].

**Fig. 5:**
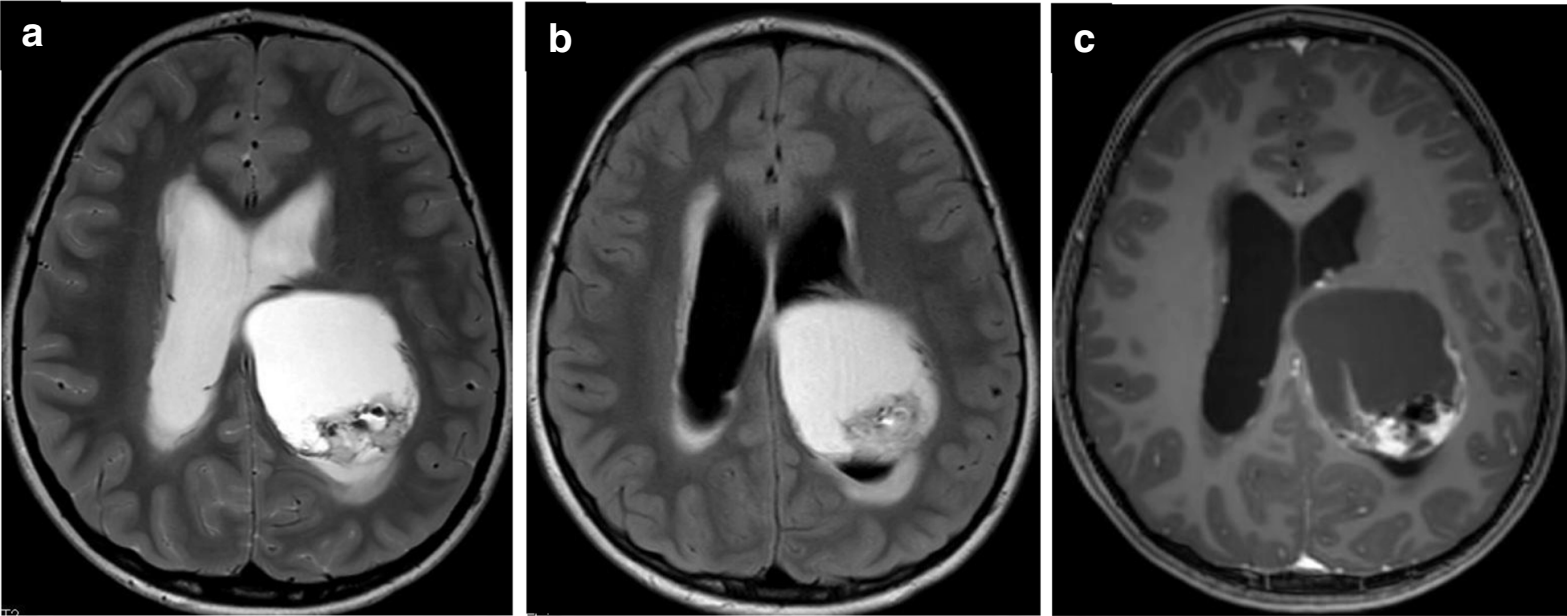
Pilocytic astrocytoma with *BRAF-KIAA* 1549 fusion in a 10-year-old male presenting with seizure. Axial T2 (**a**), FLAIR (**b**), and T1-weighted contrast-enhanced (**c**) MR images show a predominantly cystic, T2/FLAIR-hyperintense left frontoparietal periventricular tumor with a solid, avidly-enhancing mural nodule

Supratentorial pilocytic astrocytomas often demonstrate imaging characteristics similar to classic cerebellar pilocytic astrocytomas, often appearing as a well-circumscribed solid and cystic lesion with an enhancing mural nodule (Fig. 5) [44]. However, predominantly solid lesions also occur. When present, perilesional edema tends to be mild. Increased ADC values and decreased cerebral blood volumes (rCBV) can help to distinguish pilocytic astrocytomas from more aggressive supratentorial tumors such as ependymomas [44, 45]. Interestingly, an ADC threshold value of >1,189.8×10^−6^ mm^2^/s has been shown to be 93.8% sensitive and 100% specific for differentiating supratentorial pilocytic astrocytoma from pleomorphic xanthoastrocytoma [46]. MR spectroscopy may mimic higher grade lesions with elevated choline. MR perfusion typically shows rCBV values lower than contra-lateral normal appearing white matter; however, it can be variable [47].

### Pleomorphic xanthoastrocytoma (PXA)

PXAs account for less than 1% of all astrocytomas and most commonly present in children and young adults, with a median age of 20 years at presentation [45, 46, 48]. Prognosis is generally good; however, PXAs can have higher WHO grades and a propensity for anaplasia than many other LEATs [8]. As a result, some argue that PXAs should not be considered LEATs. PXAs almost always occur in the supratentorial brain, with temporal lobe involvement being most common; however, they have a propensity for CSF dissemination [49]. Common molecular genetic alterations include the *BRAF V600E* mutations and *CDKN2A/B* deletions, although mutations in *ATRX*, *FANCA/D2/I/M*, and *PRKDC* have also been reported [2, 48–51].

**Fig. 6:**
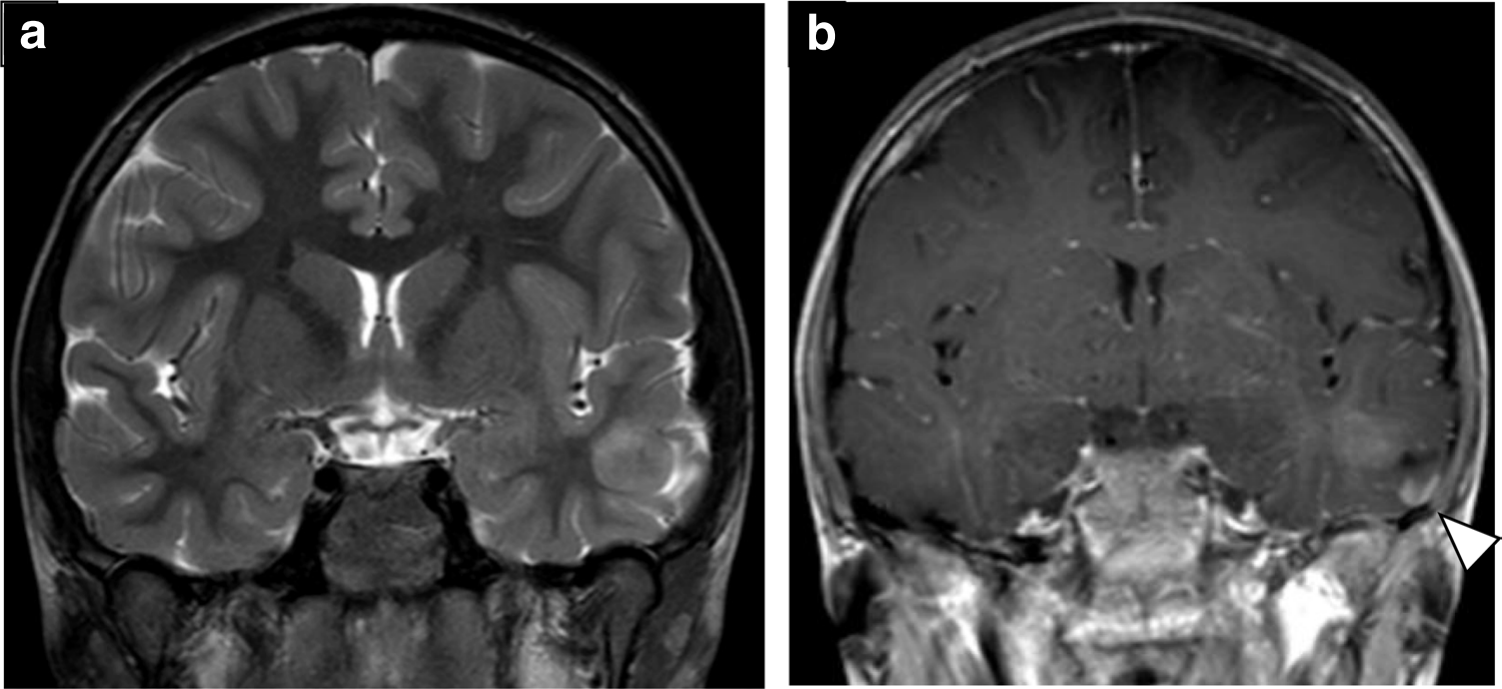
Pleomorphic xanthoastrocytoma (PXA) with *BRAF V600E* mutation and loss of *CDKN2A* in an 8-year-old male who presented with a seizure. Coronal T2-weighted (**a**) and T1-weighted contrast-enhanced (**b**) MR images show a solid, mildly expansile and T2-hyperintense mass in the left temporal lobe with faint post-contrast enhancement. Note the more avidly-enhancing focal meningeal thickening and enhancement (*arrowheads*)

The imaging appearance of PXAs is variable. They tend to present as cortically-based supratentorial tumors that may scallop the adjacent calvarium [45]. A cyst with enhancing mural nodule appearance is possible, which overlaps with the appearance of PAs, though PXAs are more likely to abut the pial surface and show adjacent meningeal thickening and enhancement (Fig. 6) [45, 49, 52]. Surrounding edema is generally minimal in lower grade lesions, while more peritumoral edema has been associated with worse prognosis in adult patients [8, 53]. Apparent diffusion coefficient values in PXA tend to be lower than those in PA, although higher than typical supratentorial ependymoma [46, 54].

## Pediatric-type low-grade gliomas

### Diffuse astrocytoma,* MYB *or* MYBL1*-altered

**Fig. 7:**
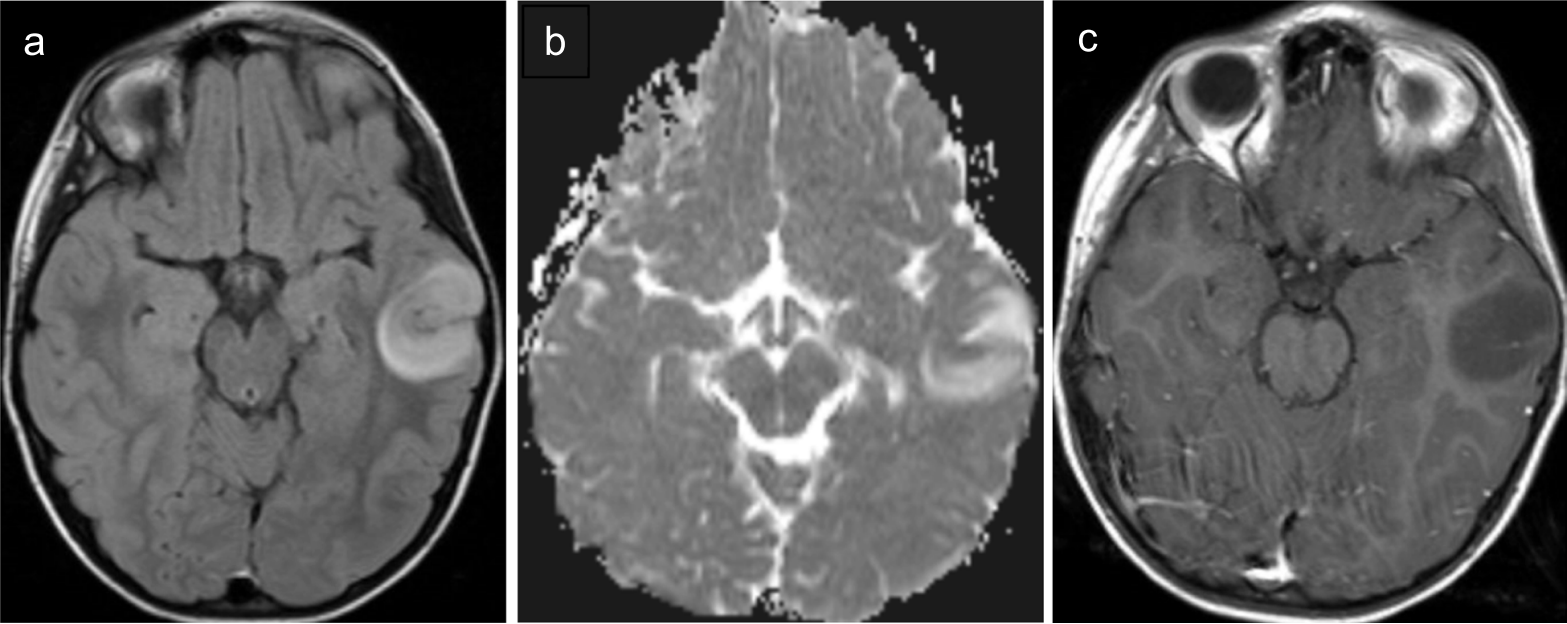
Diffuse astrocytoma *MYB/MYBL1*-altered in a 7-year-old female identified incidentally after a go-cart accident. Axial T2 FLAIR (**a**), ADC (**b**), and T1-weighted contrast-enhanced (**c**) MR images show a circumscribed, cortically-based, mildly expansile, heterogeneous, FLAIR-hyperintense left temporal mass with facilitated diffusivity and no internal enhancement

Diffuse astrocytoma-*MYB* or *MYBL1*-altered tumors were a newly recognized entity in the 2021 WHO classification of tumors of the CNS after being recognized as molecularly distinct from the closely related entity angiocentric glioma. They account for approximately 2% of pediatric low-grade gliomas and have a median age at diagnosis between 5 years and 10 years [55]. Diffuse astrocytoma-*MYB* or *MYBL1*-altered tumors most commonly arise in the cerebral cortex or subcortical white matter, although may also arise in the cerebellum or deep gray nuclei [55, 56]. As suggested by the name, amplifications and structural alterations including fusions and rearrangements in *MYB* and *MYBL1* are the typical genetic signature and are important for differentiating this indolent tumor from angiocentric glioma and the more aggressive adult-type diffuse astrocytoma [55, 57]. At imaging, diffuse astrocytoma*-MYB* or *MYBL1*-altered tumors typically present as expansile, cortically-based tumors with well-defined borders that are hypoattenuating on CT and T2-hyperintense on MRI (Fig. 7). The T2-FLAIR mismatch sign may be observed, with centrally hypointense FLAIR signal and a thin rim of peripheral FLAIR hyperintense signal [55, 58, 59]. Diffuse astrocytoma*-MYB* or *MYBL1*-altered tumors do not exhibit low diffusivity and are typically non-enhancing on post-contrast imaging; however, thin linear enhancement (with corresponding T2-hyperintensity) centrally within large tumors has been reported (“fireworks” sign) [55, 58].

### Angiocentric glioma

Initially recognized as a distinct tumor type by the 2007 WHO classification of tumors of the CNS, angiocentric gliomas are rare LEATs characterized molecularly by a *MYB-QKI* gene fusion (or, rarely, other MYB alterations) [58, 60, 61]. They occur most commonly in adolescence, though reported cases range in age from 2–83 years old [62]. Angiocentric gliomas are typically cortically-based tumors that arise most commonly in the frontal lobes; however, brainstem and basal ganglia involvement have been reported [61, 62]. Notably, brainstem presentations have a younger median age at presentation of 5 years [58]. Dual lesions co-occurring with focal cortical dysplasia occur rarely [62].

**Fig. 8:**
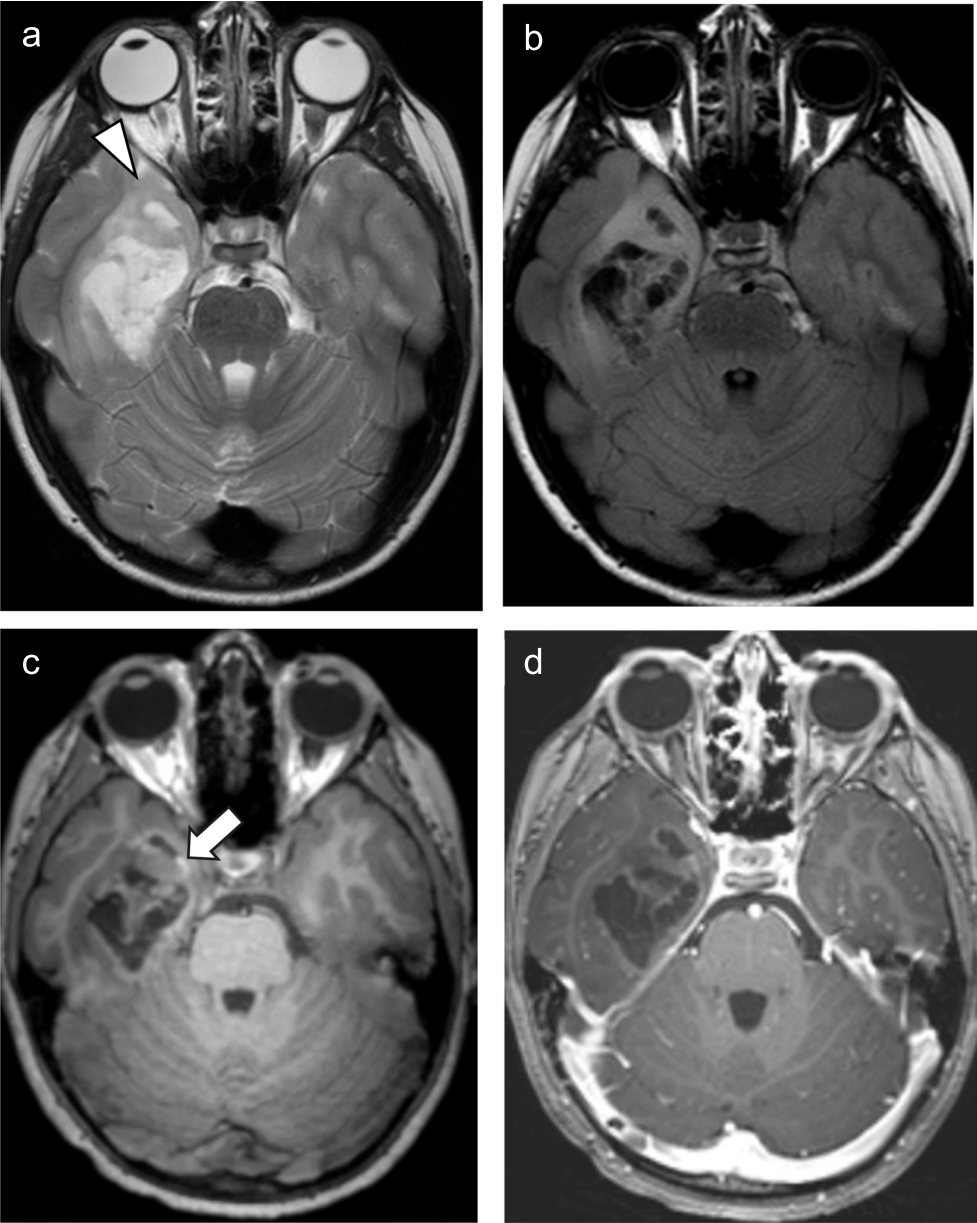
Angiocentric glioma *MYB* and *MYBL1*-altered in an 8-year-old male with a 3-year history of epilepsy. Axial T2-weighted (**a**), T2 FLAIR (**b**), T1-weighted (**c**), and T1-weighted contrast-enhanced (**d**) MR images show a non-enhancing, cortically-based tumor with subtly indistinct margins anteromedially (*arrowhead*). The T2-FLAIR mismatch sign is present and there is a thin intrinsic T1-hyperintense rim (*arrow*)

Angiocentric gliomas often present as cortically-based T2/FLAIR-hyperintense tumors that may have circumscribed or indistinct margins (Fig. 8). The T2-FLAIR mismatch sign may be observed [59]. Although they can have a somewhat variable appearance, there are a few MR imaging features that can be helpful when present. Angiocentric gliomas may show an intrinsically T1 hyperintense rim (Fig. 8) [58, 62]. Additionally, stalk-like tumoral extension toward the lateral ventricle has been reported in up to 20% of cases [62]. Contrast enhancement occurs in approximately one-quarter of cases and is most commonly heterogeneous [58]. Angiocentric gliomas do not show low diffusivity.

### Diffuse low-grade glioma (DLGG),* MAPK *pathway-altered

DLGG *MAPK* pathway-altered was a newly recognized tumor type in the 2021 WHO classification of CNS tumors and is molecularly characterized by *MAPK* pathway alterations including: *FGFR1 tyrosine kinase domain-duplicated*, *FGFR1* mutation, or *BRAF V600E* mutation [61]. These are pediatric tumors that are believed to be rare and can arise anywhere in the CNS, although most commonly occur as LEATs in the cerebral hemispheres [63]. Unlike other LEATs, DLGG *MAPK* pathway-altered tumors do not have an assigned CNS WHO grade. Maximum safe resection is the treatment of choice when feasible; however, adjuvant targeted chemotherapy is also a mainstay of therapy for many patients [58].

**Fig. 9:**
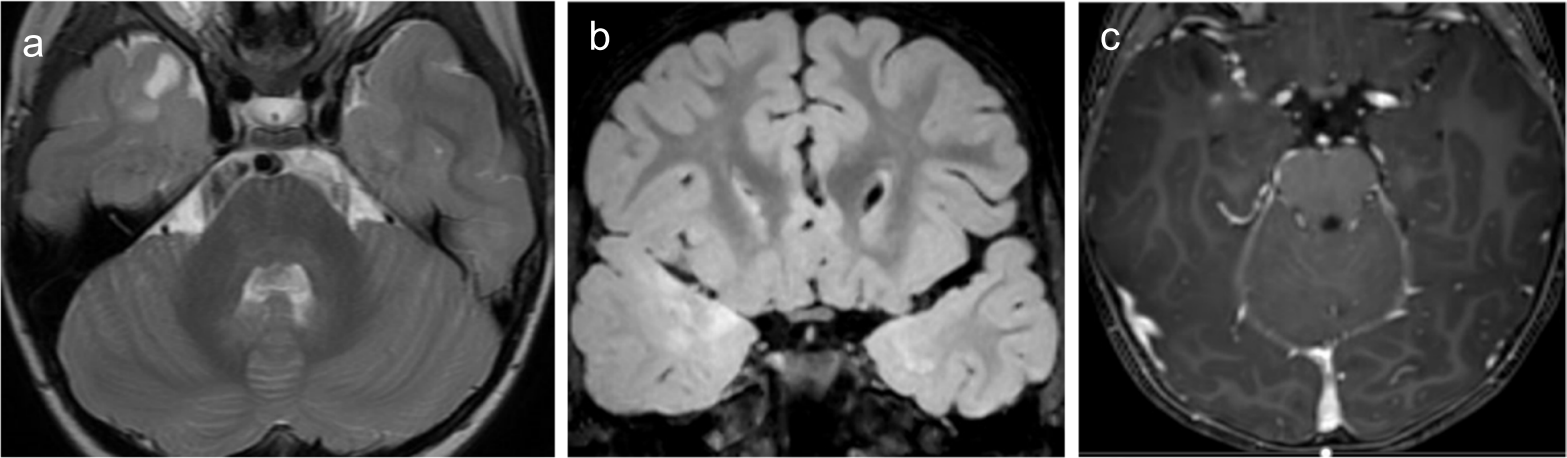
Diffuse low-grade glioma MAPK altered with *BRAF V600E* mutation in a 3-year-old male who presented with a seizure. Axial T2-weighted (**a**), coronal FLAIR (**b**), and axial T1-weighted contrast-enhanced (**c**) MR images demonstrate a T2/FLAIR hyperintense right temporal mass with indistinct posterior margins and a small nodular focus of enhancement

Literature describing the imaging characteristics for DLGG MAPK pathway-altered tumors is limited, likely at least somewhat related to tumor rarity. At MRI, these tumors are T2-hyperintense, with or without the T2/FLAIR mismatch sign, and may have well-defined or more infiltrative borders (Fig. 9) [58]. Heterogeneous enhancement and cystic components may be observed [64].

### Polymorphous low-grade neuroepithelial tumor of the young (PLNTY)

PLNTY is another newly recognized tumor type in the 2021 WHO classification of CNS tumors. Fewer than 100 cases have been reported in the literature, with a median age of 18.2 years (range 4–57) [65]. Patients most commonly present with seizures and the majority of cases occur in the temporal lobes (~65%), though they can arise in any of the cerebral lobes. Genetic abnormalities in PLNTYs typically involve the MAP kinase pathway, including *BRAF-V600E* mutations and *FGFR2/3* alterations [65, 66]. Interestingly, *BRAF V600E-*mutated tumors are associated with age of onset ≥18 years and temporal lobe location [67].

**Fig. 10:**
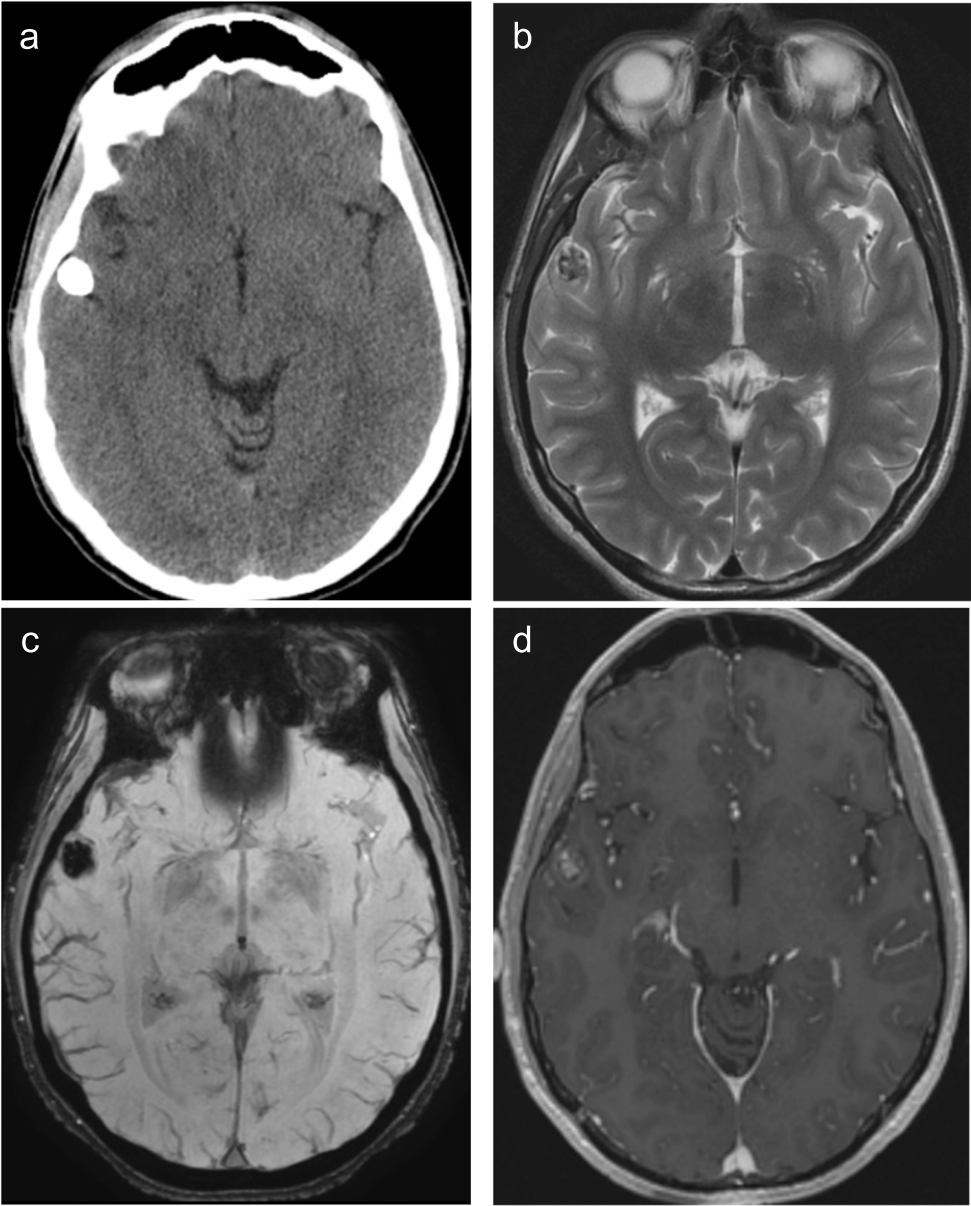
Polymorphous low-grade neuroepithelial tumor of the young (PLNTY) with *BRAF V600E* mutation in a 29-year-old male with new seizure. Axial CT (**a**) image shows a densely calcified lesion in the right temporal lobe. Axial T2-weighted (**b**), SWI (**c**), and T1-weighted contrast-enhanced (**d**) MR images reveal a circumscribed, cortically based, T2-heterogeneous signal mass in the right temporal lobe with blooming artifact (SWI) and nodular internal enhancement

PLNTY typically presents as a well-circumscribed, T1/T2-heterogeneous signal tumor in a cortical/subcortical location [68, 69]. Bulky calcifications are a helpful feature when present (Fig. 10). Small internal cysts are commonly observed, and approximately one-third of cases show post-contrast enhancement [68, 69].

## Conclusion

LEATs are a distinct group of tumors commonly encountered in pediatric drug-resistant epilepsy, necessitating surgical intervention. They are classified into three WHO categories based on histologic features and molecular genetic alterations: glioneuronal and neuronal tumors, circumscribed astrocytic gliomas, and pediatric-type low-grade gliomas. Familiarity with relevant tumor mutations and radiogenomic features of LEATs can enable radiologists to generate more accurate differential diagnoses.

## Data Availability

No datasets were generated or analyzed during the current study.
